# Novel biomarkers for neoplastic progression from ulcerative colitis to colorectal cancer: a systems biology approach

**DOI:** 10.1038/s41598-023-29344-y

**Published:** 2023-02-28

**Authors:** Mina Shahnazari, Saeid Afshar, Mohammad Hassan Emami, Razieh Amini, Akram Jalali

**Affiliations:** 1 Hamadan University of Medical Sciences, Hamadan, Iran; 2 Research Center for Molecular Medicine, Hamadan University of Medical Sciences, Hamadan, Iran; 3 Poursina Hakim, Digestive Diseases Research Center, Isfahan University of Medical Sciences, Isfahan, Iran

**Keywords:** Genomics, Biotechnology, Computational biology and bioinformatics, Systems biology

## Abstract

In recent studies, the void of evaluation and in-depth understanding of unknown clinically relevant potential molecular biomarkers involved in colorectal cancer (CRC) from the inflammatory stage of ulcerative colitis (UC) to CRC metastasis, which can be suitable therapeutic targets, is deeply felt. The regulation and interaction among different cancer-promoting molecules, including messenger RNAs (mRNAs) and micro RNAs (miRNAs) in CRC and its progression, were the aim we pursued in this study. Using microarray data, we investigated the differential expression for five datasets, including mRNA and microRNA samples related to UC, tumor/normal. Then, using robust data analysis, separate lists of differentially expressed genes (DEGs) and differentially expressed miRNAs (DEmiRNAs) were identified, which were used for robust rank aggregation (RRA) and co-expression network analysis. Then, comprehensive computational systems biology analyses, including gene ontology and Kyoto encyclopedia of genes and genomic pathway enrichment analyses, mRNA-miRNA regulatory network, and survival analysis, were employed to achieve the aim of this study. Finally, we used clinical samples to validate this potential and new target. According to this systems biology approach, a total of 98 DEGs and 8 DEmiRNAs with common differential expression were identified. By combining the distinct results of RRA and network, several potential therapeutic targets, and predictive and prognostic biomarkers for UC and CRC were identified. These targets include six common hub genes, CXCL1, CXCL8, MMP7, SLCA16A9, PLAU, and TIMP1, which are upregulated. Among these, the important and new biomarker SLC16A9 is negatively regulated by hsa-mir-194-5p, and hsa-miR-378a-5p take. The findings of the present study provide new insight into the pathogenesis of CRC in UC. Our study suggests future evaluation of the functional role of SLC16A9 and hsa-mir-194-5p and hsa-miR-378a-5p in CRC development.

## Introduction

Colorectal cancer (CRC) is the fourth most common gastrointestinal cancer in the world, and second leading cause of death by cancer. In 2020, 147,950 new cases and 53,200 deaths from CRC were estimated in the United States^1^. In recent years, the comprehensive treatments that have been applied has improved the average five-year survival of CRC only in the early stages of the disease^2^. Colitis-associated colorectal cancer (CACC) is one of the most severe complications of long-term IBD, especially ulcerative colitis (UC), and its increased risk in UC is an almost universal finding^3–5^. UC usually starts in the rectum and lower colon (sigmoid colon) but may spread continuously and involve the entire colon. As the duration of chronic intestinal inflammation increases, the risk of CRC also increases. In some studies, this annual risk rises exponentially^6^. In general, it accounts for approximately 15% of deaths among IBD patients^7^, and its incidence is 1.5–2.4 times higher in IBD patients than in the general population^7,8^. However, the incidence of IBD is increasing up to 30-fold in the last decade, and CACC is expected to grow rapidly^9^. So, UC is one of the important transition stages in the progression of CRC. Early detection and control of UC can significantly reduce the burden of CRC, as well as the incidence and economic burden caused by it. There are no reliable biomarkers that can predict the progression of inflammatory bowel disease to carcinoma. To find potential diagnostic biomarkers, and therapeutic and prognostic biomarkers, there is a need to understand the molecular pathogenesis underlying the progression of UC to malignant forms^10^. The development of high-throughput technologies and advances in molecular biology have identified new biomarkers, such as mRNA and miRNAs that are diagnostic tools for predicting the occurrence, progression, and prognosis of CRC^11,12^. And yet, in the studies of different platforms of microarray and next-generation RNA sequencing (RNA-seq), high variability and poor statistical conclusions are seen, which can be due to small sample sizes^13^. In this regard, to overcome these challenges, increasing the number of samples by considering all stages of disease progression and the new approach of robust rank aggregation (RRA) can helper^14^. In addition, gene co-expression network analysis and its robust strategy, as a powerful systems biology method, can identify potential patterns of gene connections (as modules and hub genes) between different samples based on the connectivity of gene sets. Since hub genes are not conserved between normal and disease samples, they have a significant relationship with the desired clinical feature, which can indicate their potential role in clinical, biological processes. In fact they are the golden keys, which show their capacity as candidate biomarkers or therapeutic targets^15^. Currently, studies are being conducted to increase our understanding of miRNA regulatory mechanisms and functions. MicroRNAs (miRNAs), which, by regulating the stability and translation of messenger RNA (mRNA), play a crucial role in various human diseases, especially in cancer^16^ They are regulators in various cancers, especially in CRC^15^. Since most laboratory and experimental approaches focus on identifying gene targets and investigating their physiological consequences when their expression is disturbed, introducing a systematic approach in which the pathways in which miRNAs and genes perform their actions seems very attractive^17,18^. Multidimensional analysis of omics data related to miRNAs can be helpful in identifying key therapeutic targets and prognosis of many cancers^15^. In other words, knowing the cause of differential expression of genes and their downstream targets by systems biology approaches not only leads to better diagnostic results but also lead to the design of new targeted drugs and increases the effectiveness of existing drugs^19^. The current research with a biology system approach is a combination of a data-centered model and experiments designed with this model as a center to achieve the final goal. To create a systematic and comprehensive model, we first defined a combination of critical DEGs in CRC and intestinal ulcers compared to normal tissues as specific genes. Then, by plotting the PPI network and its related analysis, the hub gene protected in all stages of CRC and shared with UC was identified. A network of important DEGs and related and common DEmiRNAs in the process of intestinal ulcers inflammation to the pathogenesis of CRC was made, aiming to determine the appropriate biomarkers of this disease, the relationship between miRNA and mRNA networks and related signaling pathways through bioinformatics. We identified potential biomarkers for diagnosis, prognosis, and therapeutic targets using computational experiments, also investigated the biological significance of hub genes in downstream processes. Among these, two candidate hub genes, SLC16A9 and CXCL8 and DE miRNA under the name hsa-mir-194-5p were further evaluated and experimentally validated to assess their biological significance and prove their biomarker capability in CRC carcinogenesis.

## Materials and methods

### Study design and data collection

**Figure 1:**
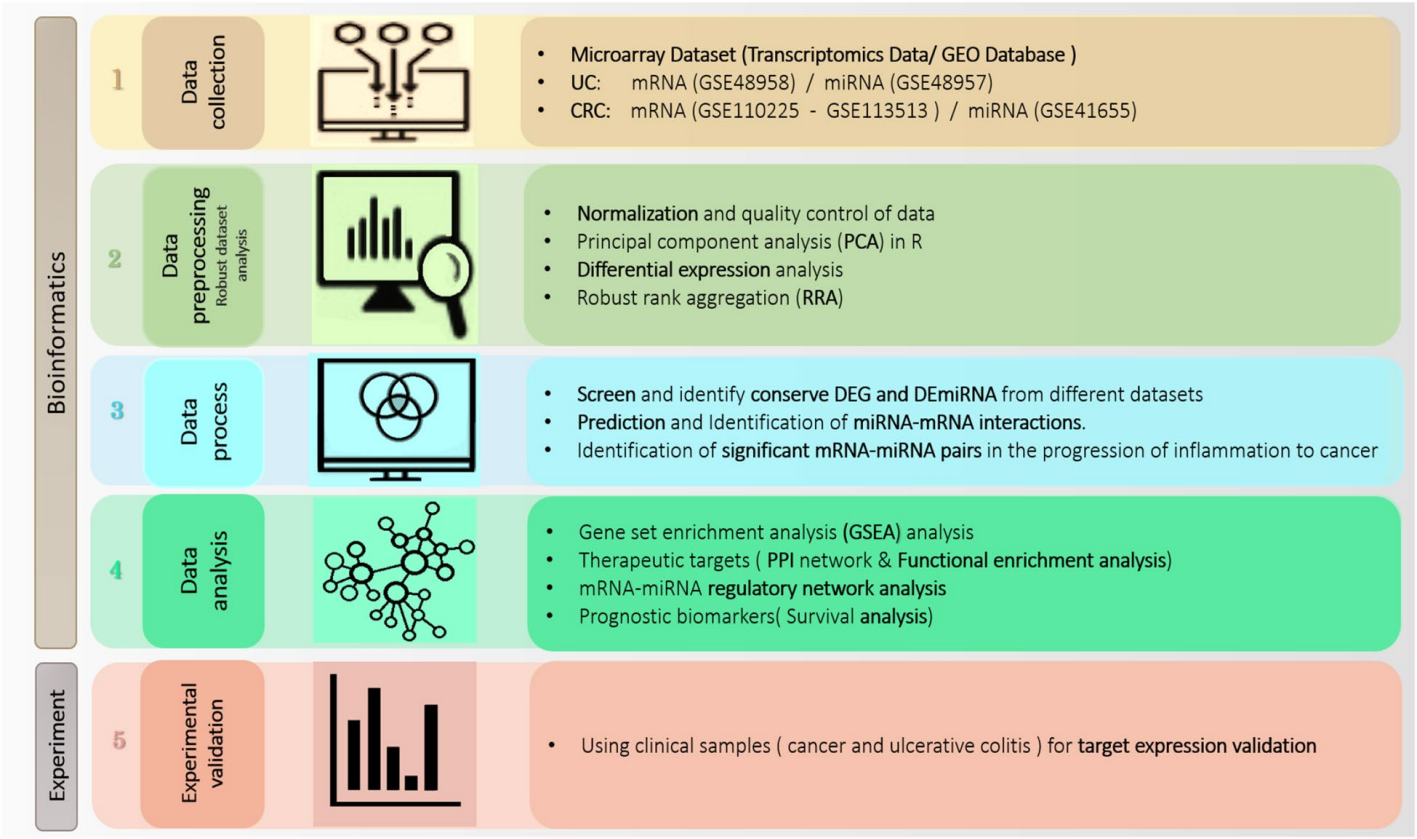
Five sets of microarray data were downloaded and analyzed for differential identification. mRNAs, miRNAs are expressed in UC, CRC and normal tissues. By performing normalization and quality control, differential expression analysis of the data was performed and then the data was entered into RRA to find. The most significant DEGs and DE miRNAs were screened in all datasets. By predicting and identifying miRNA-mRNA interactions, important mRNA-miRNA pairs were identified in the progression of inflammation to cancer. To determine the key modules shared between CRC and UC, protein–protein interaction (PPI) network analyzes were performed and functional enrichment analysis was investigated. In addition, criteria and selection of common DEGs, conserved hub genes in the progression of inflammation to cancer were also obtained. Using DEmiRNAs, DEGs as well as conserved hub genes, the mRNA-miRNA regulatory network was constructed to find potential therapeutic targets. Finally, a comprehensive downstream analysis was performed to identify potential prognostic and diagnostic biomarkers through different approaches.

The Flow chart of the present study is shown in Fig. 1. Gene expression data search in dataset format Gene Expression Omnibus (GEO) (http://www.ncbi.nlm.nih.gov/geo/), which is an array- and sequence-based applied genomic database, was conducted until 2021. Our criterion for screening GEO microarray data was based on the following materials: (1) Gene expression data were from human samples; (2) The data were from the comparison of UC/CRC samples and normal colorectal tissue; (3) The lesion and the normal tissue are from the same place; (4) The number of samples in each group was at least five or more than this number. The data includes several thousand probes from the Affymetrix (http://www.affymetrix.com) and Agilent (http://www.Agilent.com) platforms. To normalizing, R software and related packages of Bioconductor were used (Table 1).

**Table 1:** Microarray dataset.

Platform (expression array)	Number of samples		Type Data	DEGs (UR/DR)	Dataset (GEO)
	Samples (CRC/Normal)	Samples (UC/Normal)			
Affymetrix Human Gene 1.0 ST Array [transcript (gene) version]	–	21 (13/8)	mRNA	418(212/203)	GSE48958
[miRNA-2] Affymetrix Multispecies miRNA-2 Array	–	27 (17/10)	miRNA	88 (46/42)	GSE48957
[HG-U133_Plus_2] Affymetrix Human Genome U133 Plus 2.0 Array	60 (30/30)	–	mRNA	1109 (631/478)	GSE110225
Affymetrix Human Gene Expression Array	28 (14/14)	–	mRNA	2664 (1557/1089)	GSE113513
Agilent-021827 Human miRNA Microarray [miRNA_107_Sep09_2_105]	107 (92/15)	–	miRNA	55 (22/33)	GSE41655

*CRC* colorectal cancer, *UC* ulcerative colitis, *DEGS* differentially expressed genes, *UR* upregulated, *DR* downregulated.

### Unsupervised analysis (data preprocessing)

**Figure 2:**
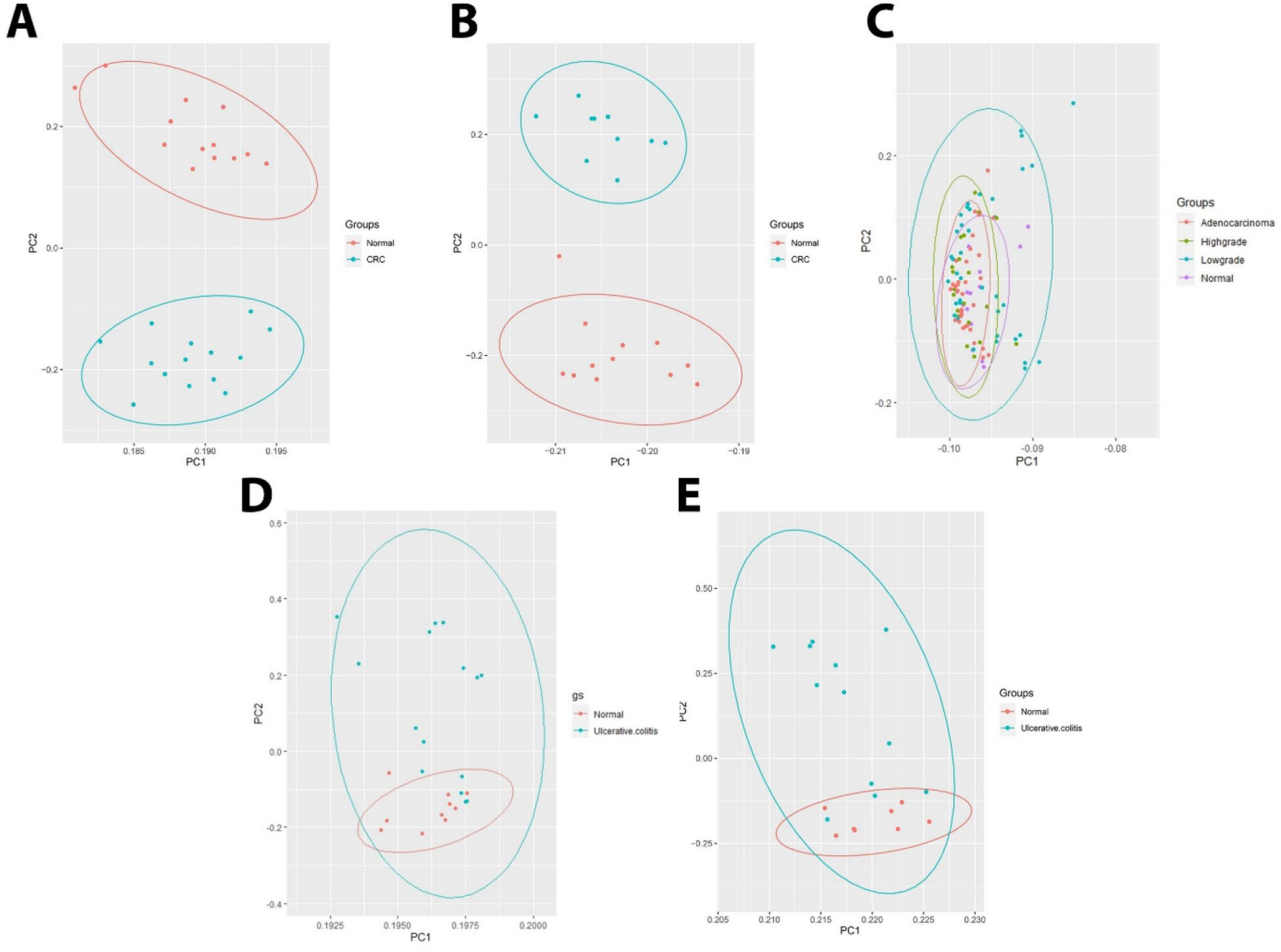
PCA diagram. (**A**) Analysis of CRC mRNA data in GSE113513 dataset. (**B**) Analysis of CRC mRNA data in GSE110225 dataset PCA. (**C**) Analysis of CRC miRNA data in GSE41655 dataset. (**D**) Analysis of UC mRNA data in GSE48958 dataset. (**E**) Analysis of UC miRNA data GSE48957 dataset.

Before DEG and DEmiRNA analysis, quality control of the normalized data set was performed using principal component analysis (PCA) to identify outliers without biological relevance and to remove them (Fig. 2). For those genes that have more than one probe ID, the average expression was considered for gene expression value. The LIMMA R package (version 4.1.0) was used to identify statistically significant DEGs and DEmiRNAs between UC/CRC and adjacent normal tissue samples in the GEO microarray dataset^20^.

### Supervised analysis (identification of DEmiRNA & DEGs)

**Figure 3:**
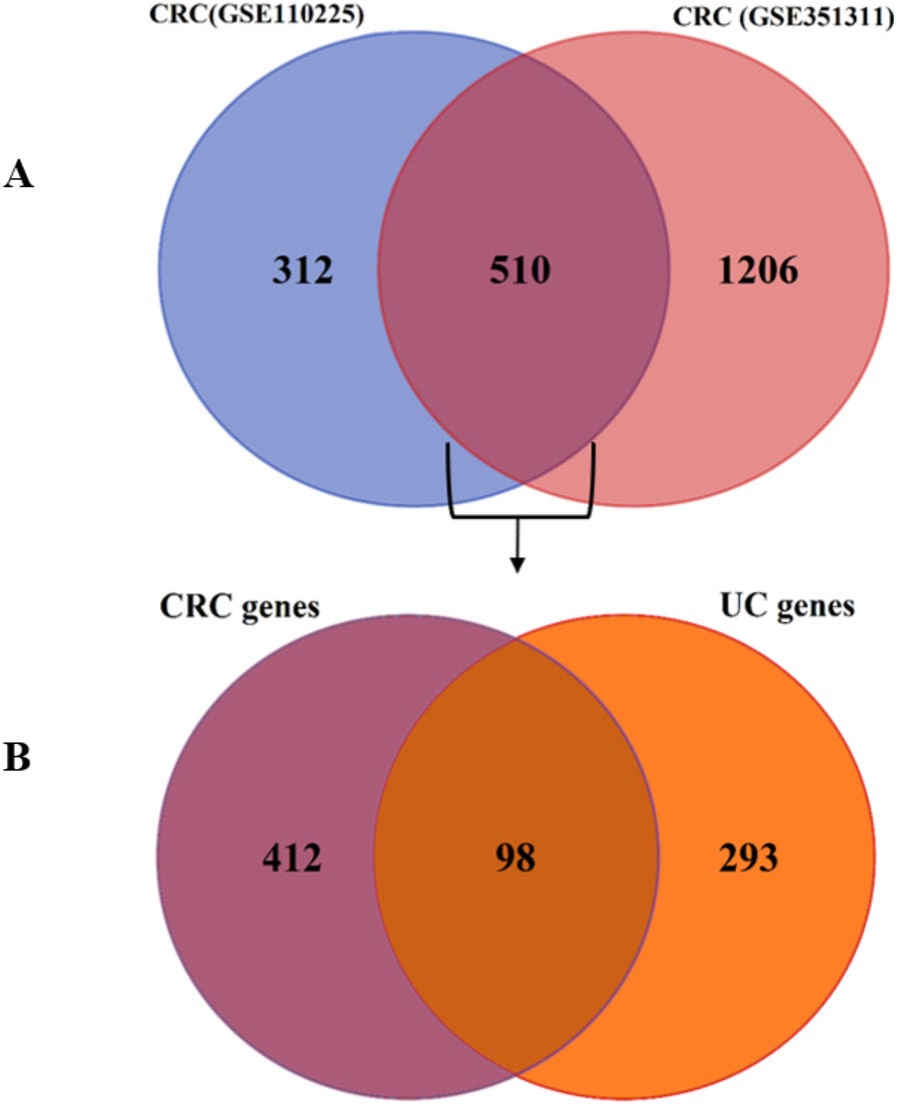
VENN diagram between CRC and UC related mRNA datasets. (**A**) VENN diagram between two datasets related to CRC mRNA, which finally obtained 510 common DEGs. (**B**) VENN diagram between the results of the VENN diagram of part A and the dataset related to UC mRNA, which finally obtained 98 common DEGs for the analysis of the next steps.

**Figure 4:**
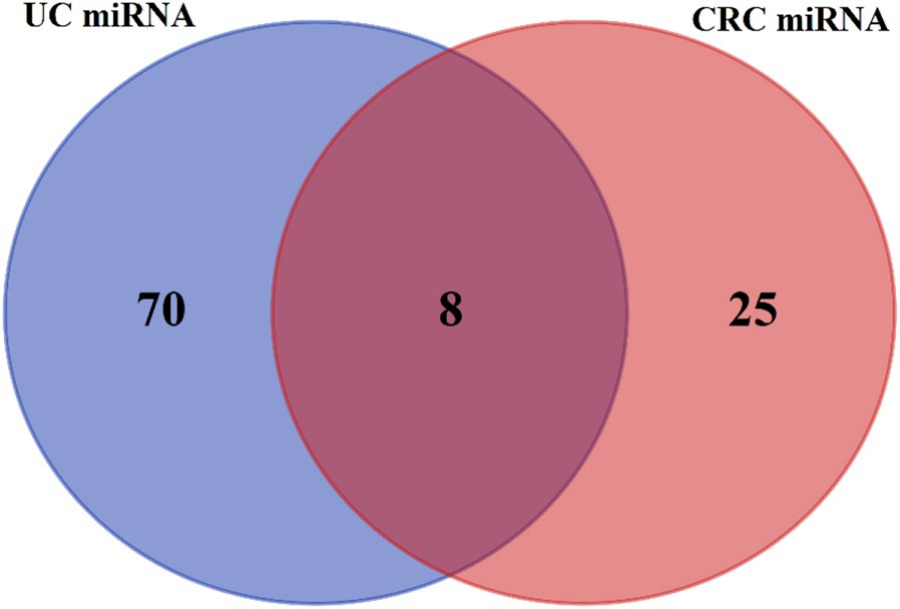
VENN diagram between miRNA datasets related to CRC and UC. The VENN diagram between the two data sets related to miRNA CRC and miRNA UC, finally 8, common miRNA DE were obtained.

We used the edgeR^21^ and LIMMA to filter and identify significant DEG and DEmiRNA packages between tumor and inflammatory samples. In this regard, low-expression mRNAs and miRNAs were removed using the edgeR package. The trimmed mean m-values (TMM) normalization method was used to remove hybridization biases between libraries. Variance modeling at the observation level (VOOM) function from the LIMMA package was also used to convert read counts to log CPM (logs per million). Statistically significant DEGs for GEO microarray data based on cut-off criteria as false discovery rate (FDR) threshold < 0.05 and | Log2 fold change (FC)|> 1 were evaluated. Also, miRNAs with FDR value < 0.05 and |log2(FC)|> 0.5 were considered as differentially expressed molecules (DE miRNAs). We applied the RRA algorithm (R package) to merge different lists of DEGs into a final strong list of DEGs and DEmiRNA. Accordingly, the results of each data set were ranked according to the FC value of each gene. So, to identify robust DEGs, RRA analysis was performed under a cutoff point of FDR < 0.05. Finally, we used the VENN diagram to extract common and conserved DEGs and DEmiRNAs between UC and CRC datasets (Figs. 3 and 4).

### miRNA-mRNA interaction analysis

Prediction of miRNA targets selected in the previous step was made based on miRNA target prediction databases, which include: miRTarBase (https://mirtarbase.cuhk.edu.cn/), miRWalk (http://mirwalk.umm.uni-heidelberg.de/), miRDB (http://www.mirdb.org/), and miRanda (http://www.microrna.org/microrna/home.do). In this regard, the interactions between mRNA-miRNA that existed in all four databases were used as the final target pair for downstream analysis. Each of these databases uses different algorithms to predict gene targets and prioritize the predicted genes based on the score they get. In this study, the targets of miRNAs were finally selected based on the following: 1- Being identified by the above databases (at least 3 out of 4 software) and having the maximum prediction score. 2-Having a role in the pathways related to the pathogenesis of CRC and UC. 3- Having expression changes in CRC and UC based on previous studies.

### Gene set enrichment analysis (GSEA)

GSEA is used to identify classes of miRNAs and mRNAs that are overrepresented in a large set of genes or proteins and may be associated with disease phenotypes. To screen miRNA and mRNAs with differential expression between different disease groups compared to the control, this analysis is performed. In this analysis, screening parameters have considered as FDR < 0.25 and 1000 random permutations.

### Analysis of PPI & mRNA-miRNA network

To determine common hub genes of UC and CRC, first, we drew a PPI network of screened mRNA (DEGs) by means of STRING (https://string-db.org/) and Cytoscape tools and identified key genes and related modules. To identify the most important DEGs with high degrees as therapeutic targets, we used the clustering algorithm of molecular complex detection (MCODE) Score 8.5 and Cytohubba in Cytoscape software, and processed data with default parameters^22,23^. So, the central and superior DEGs that were shared between the centrality, degree, MCC, MNC, betweenness, interconnectivity, and closeness parameters of the network were chosen as the final targets and hub gene^24,25^. Eventually, the analysis of mRNA-miRNA networks was performed, to further investigate the molecular mechanisms and critical regulators in the CRC pathogenesis. In this regard, we identified mechanisms in the progression of the disease, and determined the relationships between hub genes and key miRNAs based on the role and interaction with each other.

### Analysis of biological pathways and functional enrichment analysis

To analyze the biological pathways and show the primary biological actions, the main potential targets of hub genes conserved and shared between CRC and UC, and also to reduce the complexity of Gene Ontology (GO) and Kyoto Encyclopedia of Genomes (KEGG) analysis (www.kegg.jp/kegg/kegg1.html)^24^ was used. Biological process (BP), cellular component (CC), and molecular function (MF) potential targets were clustered based on R packages ClusterProfler and GO plot in R software (version: 3.12.0)^26^. ClusterProfler results were shown in a dot plot, where the enriched pathways were presented using the gene ratio, adjusted *p*-value, and count. Chord diagrams depict the relationship between conserved hub genes and GO terms (biological process, cellular component, molecular function), and KEGG pathways. Expressions with adjusted *p* < 0.05 were considered statistically significant.

### Survival analysis

The Cancer Genome Atlas (TCGA) database and Survival and Survminer packages were used to investigate the relationship between the screened biomarkers and the survival rate of CRC patients. The results are displayed in the form of a Kaplan–Meier survival rate chart. Also, the survival analysis of the selected biomarker with the most significant effect on the mechanisms involved in pathogenesis is done based on the target genes by the TCGA database.

### Patients

A total of 30 tissue samples were collected from Iranian people referred to Poursina Hakim Research Institute in Isfahan. Patients were suffering from different stages of CRC and UC during 2021–2022. The study protocol was approved by the ethics committee of Hamadan University of Medical Sciences (ethical code: IR.UMSHA.REC.1400.123). None of the selected patients had undergone different treatments, such as chemotherapy or radiotherapy, before sample collection. In addition to each tumor and inflammatory sample, a sample of adjacent healthy tissue was collected as a control sample. All samples were stored at − 80 °C until downstream expression analysis.

### Real-time quantitative PCR assay

Total RNA was isolated from tumor, inflammatory and healthy tissues using the RNX-Plus kit (CinnaGen, Iran) and converted into complementary DNA (cDNA) using the RevertAid first-strand cDNA synthesis kit (Thermo Fisher Scientific, USA). Quantitative reverse transcription PCR (RT-qPCR) was performed in duplicate for each sample based on SYBR Green and a Real-Time PCR Detection System LightCycler 96 (Roche, USA) according to the instructions.

For RT-qPCR, CXCL8 primers (Sinaclon, Iran) were 5′CAAGAATCAGTGAAGATGCCAGTG-3′ (sense) and 5′- CAACCCTACAACAGACCCACAC-3′ (antisense). For RT-qPCR, primers SLCA16A9 (Sinaclon, Iran) were: 5′-TTGCTGAGATGTGGGTGAAC-3′ (sense) and 5′-AGAACGGAATGATTGAGAAATGTG-3′ (antisense). GAPDH primers (Sinaclon, Iran): 5′-′AAGGCTGTGGGCAAGGTCATC-3′ (sense) and 5′-′GCGTCAAAGGTGGAGGAGTGG-3′ (antisense). miR-194-5p and U6 snRNA primers were purchased from Ana cell (The primers sequence is reserved with the company itself.). The 2-△△Ct method (Livak’s formula for calculating fold change) was used to calculate the relative gene expression level of SLCA16A9, CXCL8, and GAPDH. Their levels were normalized to GAPDH mRNA level, For miRNA, U6 snRNA was used as reference. In addition to RT-qPCR, we could also validate our results using a homogenous clinical data set with more samples.

### Statistical methods

Statistical analyzes were performed using the R programming language and Graphpad Prism version 9.0 (Graphpad Software). All differences between the two groups were analyzed by using the Tukey post hoc test, and differences between different groups were analyzed by Kruskal–Wallis test. The Kolmogorov–Smirnov test was used to check the normality of data distribution. The correlation test between statements was evaluated by calculating Pearson correlation coefficients. For all statistical tests, *P* < 0.05, *P* < 0.01, and *P* < 0.001 were considered statistically significant.

### Ethical approval and consent to participate

All procedures were performed in accordance with the Declaration of Helsinki and approved by the ethics committee of the Hamadan university of medical sciences (ethical code: IR.UMSHA.REC.1400.123). Informed consent was obtained from all subjects and or their legal guardians. Patient samples were collected from Poursina Hakim Research Institute (Esfahan, Iran).

## Results

### Data collection and analysis

The raw data of the microarray expression profile, related to the differential expression of miRNA and mRNA, including samples of tumor and healthy tissue of CRC patients and inflammatory and healthy tissue of UC patients, belonging to the dataset format from the GEO database with accession numbers GSE41655, GSE113513, GSE110225, GSE48957, GSE48958 were obtained (Table 1). We use principal component analysis (PCA) was used as an unsupervised analysis to identify outlying samples without biological significance (Fig. 2). This analyze used for the data distribution of the received datasets between different samples and reduce the volume of data and visualize the data. The RRA package in R was used to perform data set integration and analysis to obtain robust DEGs with FDR < 0.05 and logFC > 1 and for robust DEmiRNAs with FDR < 0.05 and logFC > 0.5. In CRC, 510 common DEGs were identified using Venn analysis (Fig. 2), of which 98 common DEGs were identified between UC and CRC. Finally, 98 final and shared DEGs between UC and CRC were extracted (Figs. 2 and 3), which 64 DEGs were downregulated and 28 DEGs were upregulated. Also, the expression of 6 genes in UC and CRC was regulated in a contradictory manner. They were used and analyzed for the next stages of analysis. This process was also used similarly to obtain and extract DE miRNAs (Fig. 4 and Table 2), where eight common DEmiRNAs were identified between CRA and CRC.

**Table 2:** Log fold changes and active *P *value and FDR are related to DEmiRNA. DE miRNAs in CRC and UC conditions are common and shared in dataset II. miRNAs with FDR < 0.05 and |log2(FC)|> 0.5 are considered as differentially expressed molecules (DEGs).

DEmiRNAs	Dataset I			Dataset II		
	CRC			UC		
	logFC	*P* value	FDR	logFC	*P* value	FDR
hsa-miR-342-3p	− 0.92	0.00	0.00	− 0.64	0.00	0.02
hsa-miR-194-5p	− 1.56	0.00	0.00	− 1.12	0.00	0.00
hsa-miR-375-3p	− 1.97	0.00	0.00	− 1.52	0.00	0.00
hsa-miR-141-3p	− 1.77	0.00	0.00	− 1.96	0.00	0.00
hsa-miR-378a-3p	− 1.28	0.00	0.00	− 1.92	0.00	0.00
hsa-miR-378a-5p	− 0.59	0.00	0.00	− 1.94	0.00	0.00
hsa-miR-320d	− 0.80	0.00	0.00	− 0.58	0.00	0.00
hsa-miR-150-5p	− 0.84	0.00	0.00	1.40	0.00	0.00

### Gene set enrichment analysis (GSEA)

**Figure 5:**
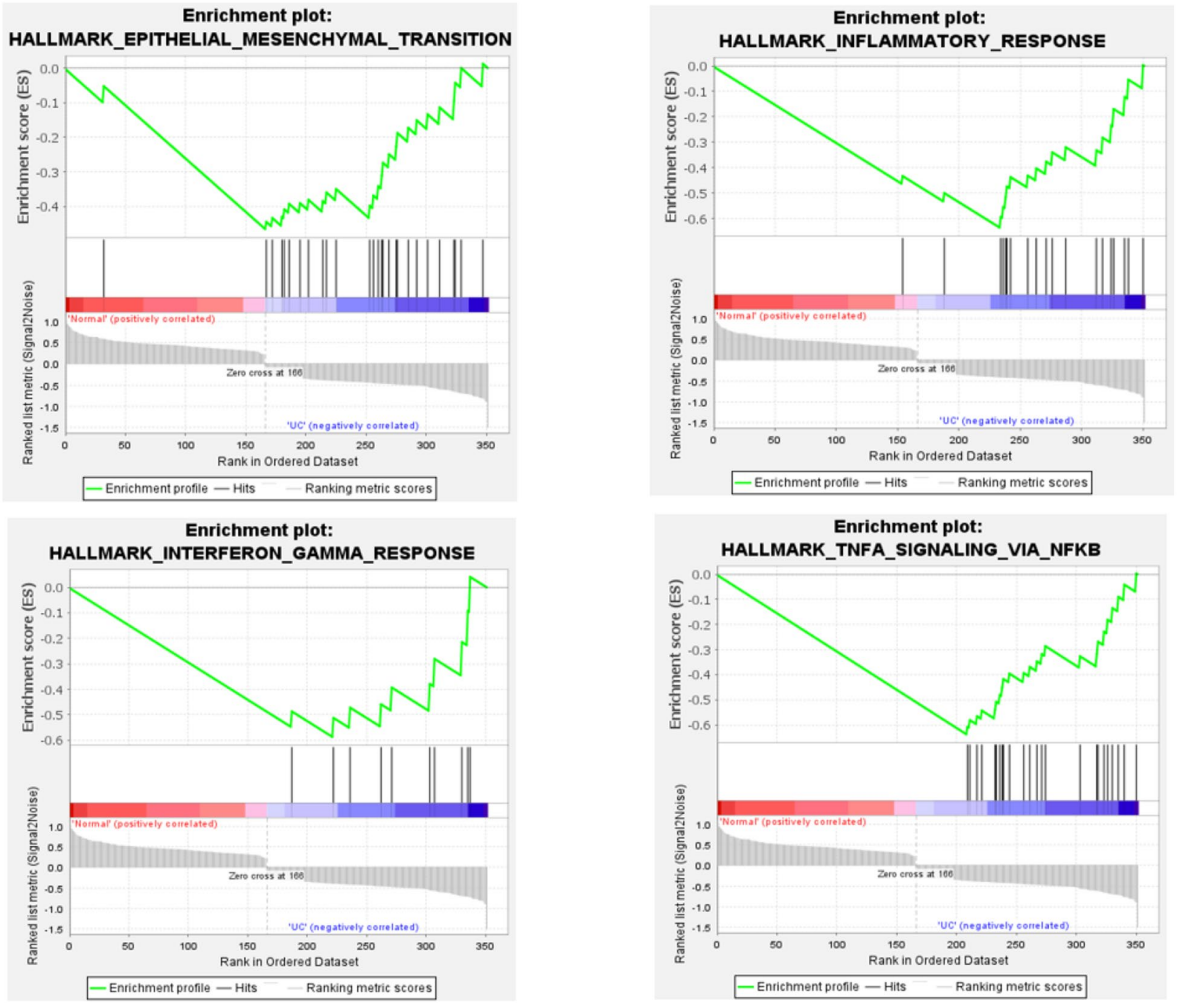
The results of GSEA analysis for DEGs with differential expression in UC compared to control.

**Figure 6:**
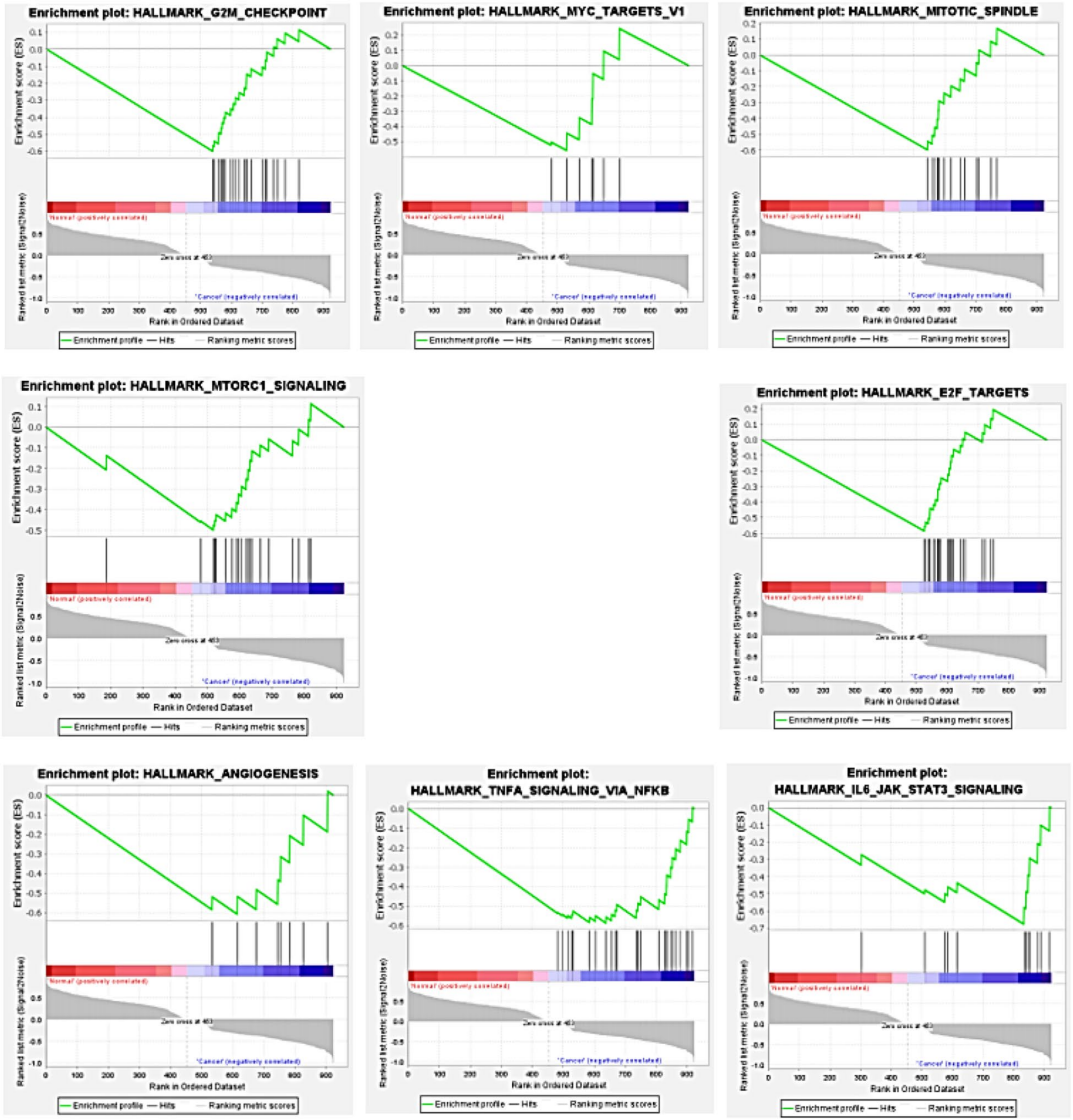
Results of GSEA analysis for DEGs with differential expression in CRC compared to control.

In the screening of DEGs that play a role in the progression of inflammation to cancer, nine pathways were found to be significant for inflammation pathway genes. Among these pathways, four important and common pathways are highly correlated with UC, include: inflammatory pathways, Epithelial-mesenchymal transition, response to interferon-gamma (IFN-γ), and angiogenesis. Also, this analysis had 8 critical pathways out of 15 significant pathways in CRC, which are mainly related to MYC-targets, MTORC1 signaling, Angiogenesis, G2M checkpoint, E2F targets, IL6_JAK_STAT3, Mitotic spindle, and TNFA signaling pathways. In this analysis, screening parameters are FDR < 0.25 and 1000 random permutations. Figures 5 and 6 show the results of this analysis for UC and CRC, respectively.

### Identification of hub genes and analysis of their functional enrichment

**Figure 7:**
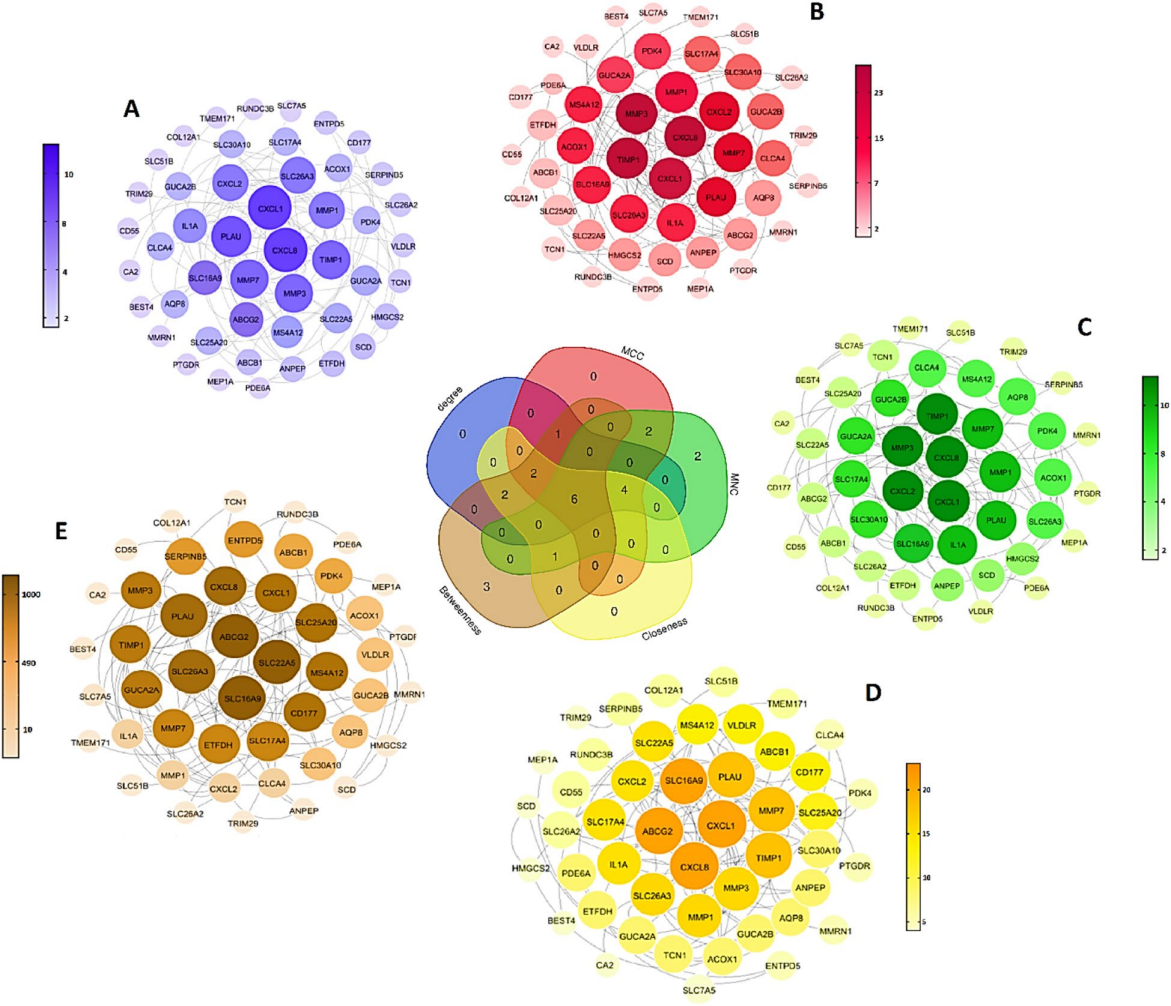
PPI Networks analysis, The hub genes were identified according to VENN diagram for result of cytoHubba and MCODE analysis. Modules were shown from the center to the outside of the network according to their score and importance by size and color index. (**A**) The highest  Degree score of the cytoHubba algorithm and a score of 8.5 MCODE (**B**) The highest score of the MCC of the cytoHubba algorithm and a score of 8.5 MCODE (**C**) The pattern of the highest score of MNC of the cytoHubba algorithm and a score of 8.5 MCODE (**D**) the highest score of the Closeness of the cytoHubba algorithm and a score of 8.5 MCODE.  (**E**) The highest score of the Betweenness of the cytoHubba algorithm and a score of 8.5 MCODE.

**Figure 8:**
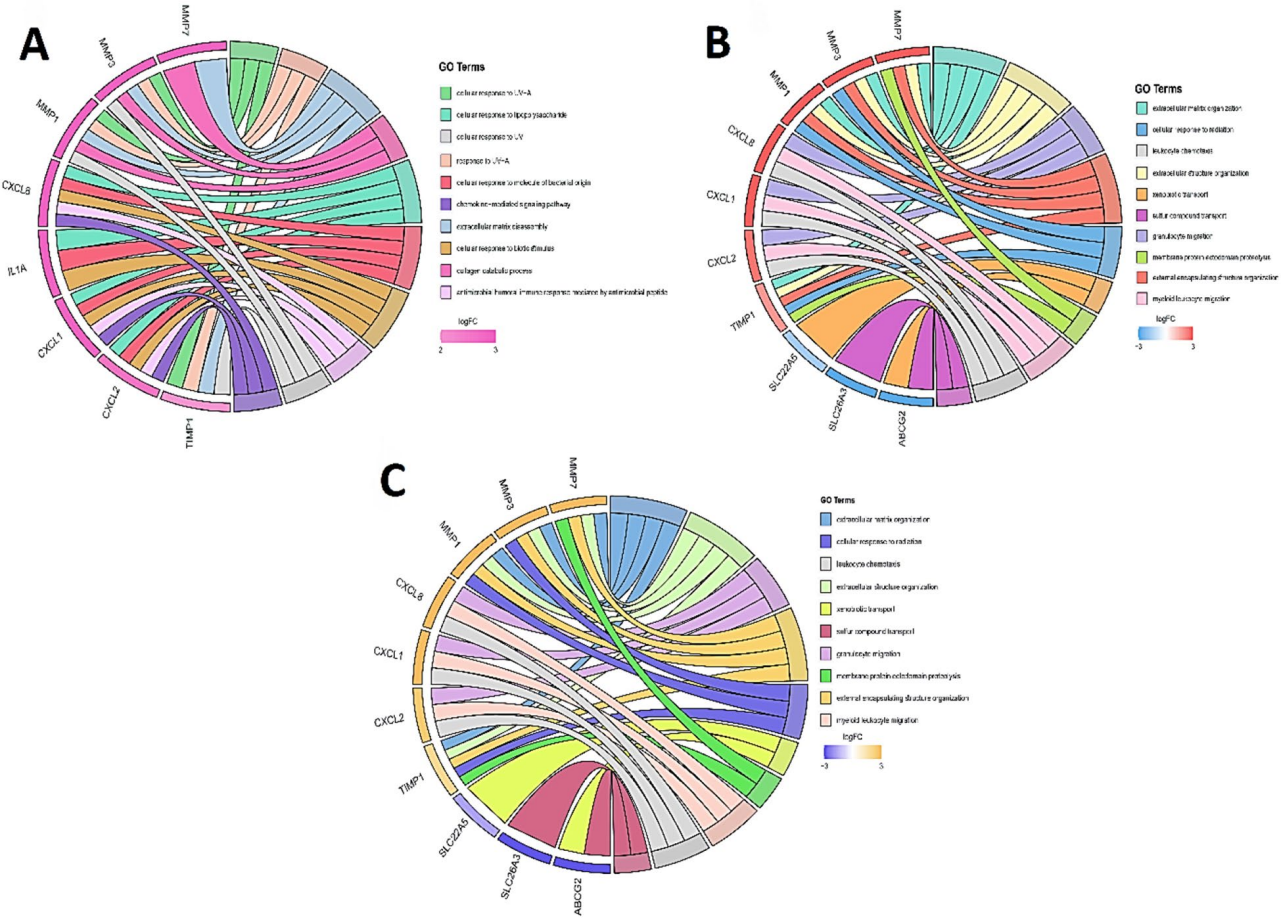
Gene Ontology (GO) cluster diagram showing the chord dendrogram of hub genes expression spectrum clustering. (**A**) Hub genes expression spectrum clustering based on biological process (BP). (**B**) based on the cellular component (Cellular Component: CC). (**C**) based on molecular function (Molecular Function: MF).

**Figure 9:**
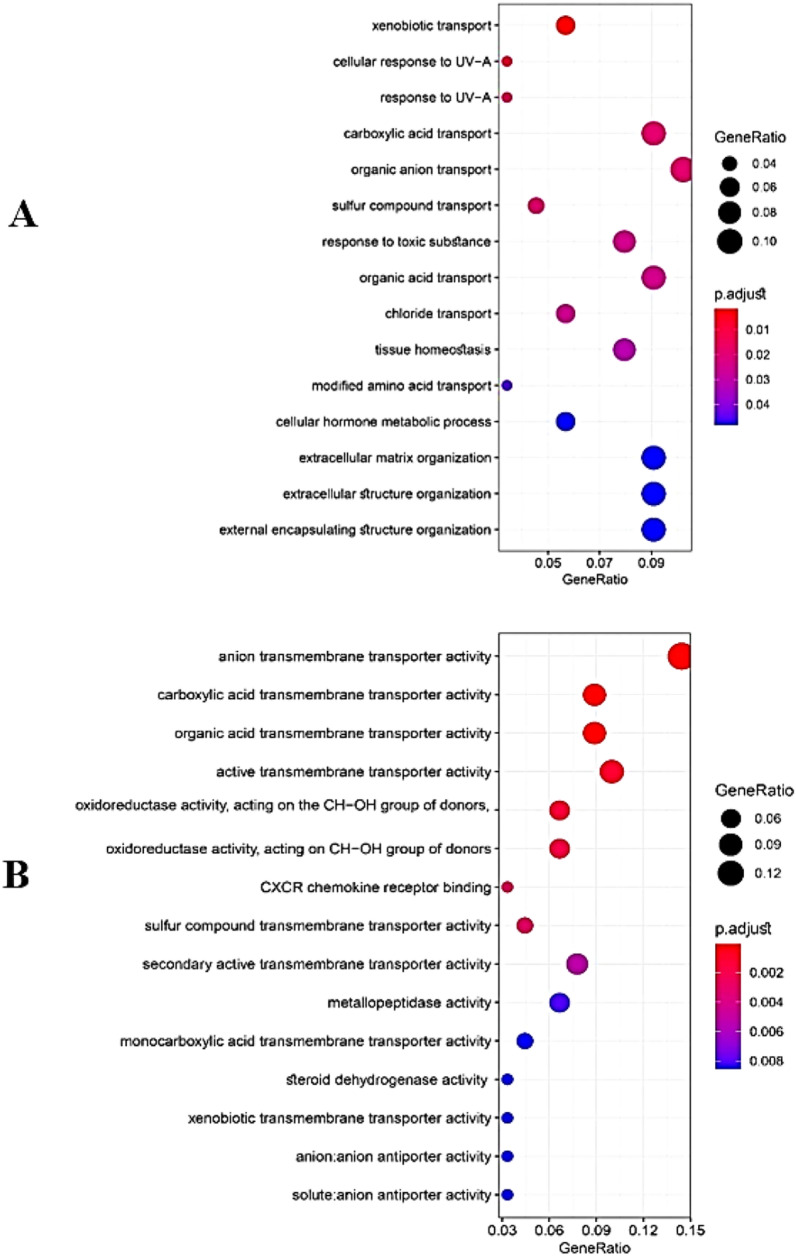
Bubble plot chart of Kyoto Encyclopedia of Genes and Genomes (KEGG) Enrichment path analysis of overlapping DEGs by ClusterProfiler and Rplot packages. The y-axis represents the KEGG-enriched terms. The x-axis represents the gene ratio. Dot size indicates the number of genes under a particular term. The color of the dots indicates the FDR value. (**A**) BP biology pathways (**B**) molecular function.

**Figure 10:**
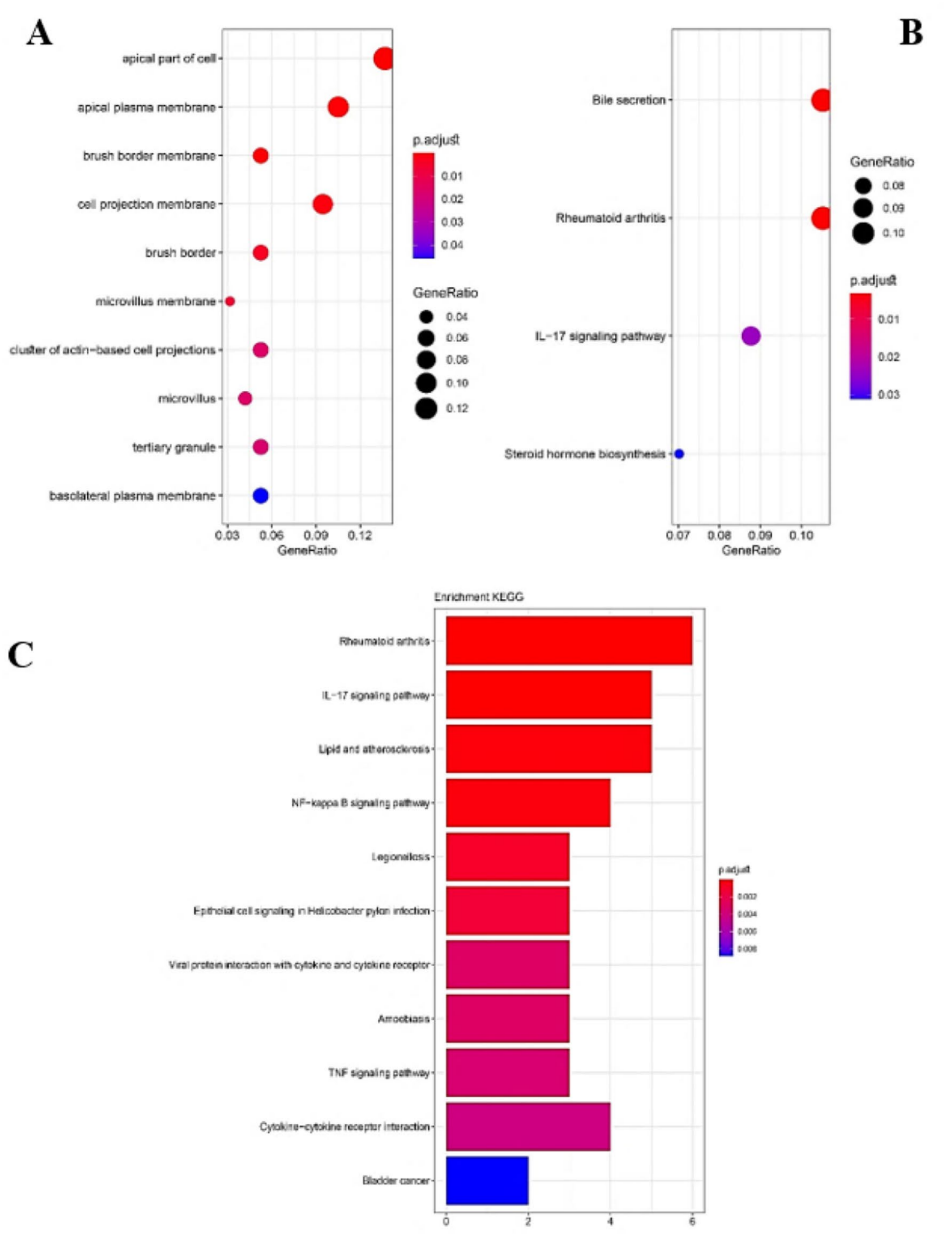
Bubble plot and bar chart analysis (KEGG) analysis of overlap DEG enrichment pathway by ClusterProfiler and Rplot packages. (**A**) Cellular component (CC) (**C** and **B**) pathways of pathogenesis.

We entered the obtained common DEGs into the STRING database to construct the PPI network and to determine the common hub genes of UC and CRC. Interaction information was obtained (high confidence = 0.7), and used Cytoscape software to visual analysis. Finally, PPI networks were constructed with 47 nodes and 96 edges. Nodes without interfaces were removed. In these networks, 16 nodes were URs, and the rest were DRs, except MMRN1, which was inconsistently up and down set in the three datasets. The networks were analyzed to determine common hub genes, and 47 nodes were visualized and sorted from inside to outside according to the degree score (Fig. 7A–E). Ultimately, according to the sharing of the results of Cytohubba, and considering the results of MCODE analysis, six central nodes, were identified including: CXCL8, CXCL1, SLCA16A9 PLAU, TIMP1 and MMP7. As visualized by a Venn diagram, six candidate genes were defined as hub genes (module genes) and DEGs in both CRC and UC. These were in the highest scores of Degrees, MCC, MNC, Betweenness, and Closeness. These genes that were upregulated in both the CRC and the UC, involved in UC inflammation and CRC progression (Fig. 7). For gene hub functional enrichment analysis, DAVID was used to discover significant GO enrichment terms (including biological process (BP), cellular component (CC), and molecular function (MF)) and KEGG pathway analysis separately (Figs. 8 and 9). The results indicated that the hub genes were significantly enriched in specific CC, which included the organization of the external structure of encapsulate and extracellular matrix, migration of leukocytes and myeloid, plasma membrane, extracellular region, and cellular response to radiation (Fig. 8B). Genes were mainly enriched in proteolysis, response to external stimuli, and UV. In the BP group, the most important processes of UC and CRC were mainly identified in the catabolic process of collagen, antimicrobial humoral immune response, response to external stimuli, separation of extracellular matrix and biotic stimulus, etc (Fig. 8A). Genes involved in the organization of the extracellular matrix, external encapsulating structure, migration of granulocytes and myeloid leukocytes, membrane protein proteolysis, etc. were among the richest GO MF terms (Fig. 8C). In the KEGG pathway, genes were mainly involved in transporters and extracellular structures. They were also generally enriched in inflammatory diseases, rheumatoid arthritis, and bile secretion. Based on KEGG pathway analysis, these hub genes were mainly associated with membrane transporter and oxidoreductase activity, bile secretion, extracellular structures, etc. (Figs. 9 and 10).

### Identification of therapeutic targets based on mRNA-miRNA and PPI networks

To further investigate the mechanisms involved in disease pathogenesis and the most important regulators of these mechanisms involved in disease progression, a shared mRNA-miRNA network was constructed based on the results of edgeR/LIMMA package analysis. As mentioned earlier, we identified a total of 8 DE-miRNAs commons to UC and CRC in databases by comparison with adjacent normal tissue samples. Among the 98 DEGs and 8 DE-miRNAs in joint, there were a total of 11 reliable miRNA-mRNA pairs, based on the result of prediction databases. These pairs were used for further analysis (Fig. 7).

**Figure 11:**
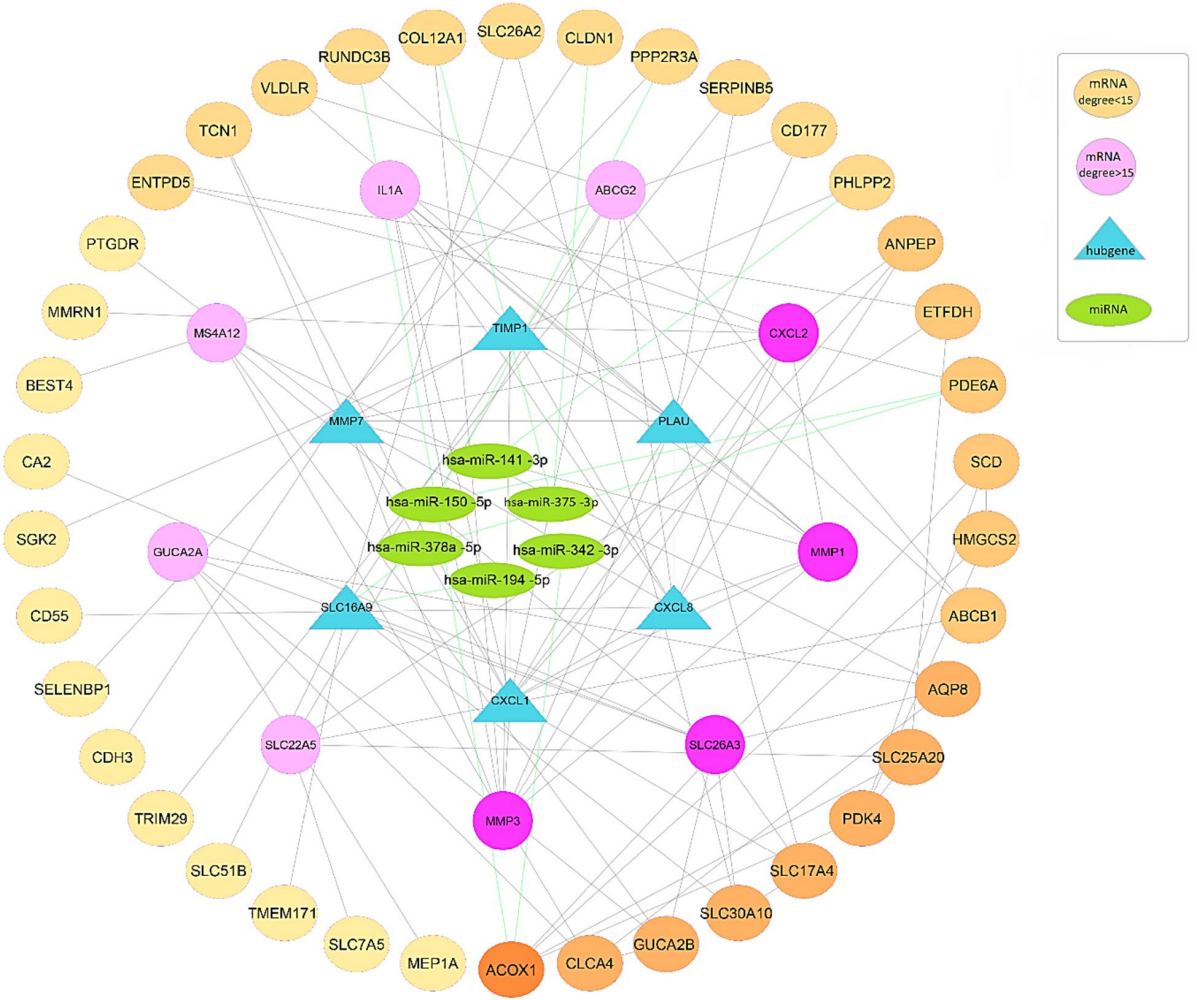
Visualization of miRNA-Gene-Network using gene expression data and interactions predicted by STRING and Cytoscape tools. Circles and triangles represent genes and ovals represent miRNAs. Key genes in the network always have the highest rank. The outer circles are the genes whose rank and degree are less important in the network analysis and related algorithms, whose importance and degree in terms of involvement in the pathogenesis pathways are shown in pale yellow to orange color degrees. Pink circles and blue triangles are 15 key genes in terms of Drgree, Betweenness, and Closeness, in other words, the center of the network indicates the degree. And the gradation of pink circles is shown as a color plot from light to dark pink. The blue triangles represent six important hub genes in terms of network analysis and MCODE and Cytohubba algorithms, according to the degree of importance. Green links indicate the target score between the miRNA and the target gene.

It showed the complex interaction of different miRNA-mRNA pairs, which among the six DE-miRNAs shared more edges with hsa-miR-150-5p. After that, hsa-miR-375-3p and hsa-miR-378-5p have the most interaction in the co-expression network of CRC and UC, which generally includes marginal DEGs (with low score and degree). Among these, miRNAs hsa-miR-378a-5p and hsa-miR-194-5p interact with the centrality of the interactive network and regulate high-order nodes, or in other words, they have a common edge with hub genes. In addition, SLCA16A9, ACOX1, PDE6A have a potential interaction with DEmiRNA, and PPP2R3A, CLDN1, COL12A1, PHLPP2 also interact with DEmiRNAs, which may indicate their vital role in CRC carcinogenesis. in the other word, the centrality of the network (hub genes), and interactions with them form an essential part of the analysis path. Also, in some recent studies, the important role of miR-378a-5p in the initiation and progression of cancer or UC disease has been pointed out through different mechanisms^27–30^. Accordingly, we have focused on the importance, novelty, and ability to directly target hub genes (DEGs) in the regulatory network of UC progression to CRC carcinogenesis, on network centrality and a higher degree of connectivity among nodes rather than miRNA. Among these, SLCA16A9, CXCL8, and hsa-miR-194-5p were selected for further evaluation (Fig. 11).

### Identification of biomarkers with potential prognostic value

**Figure 12:**
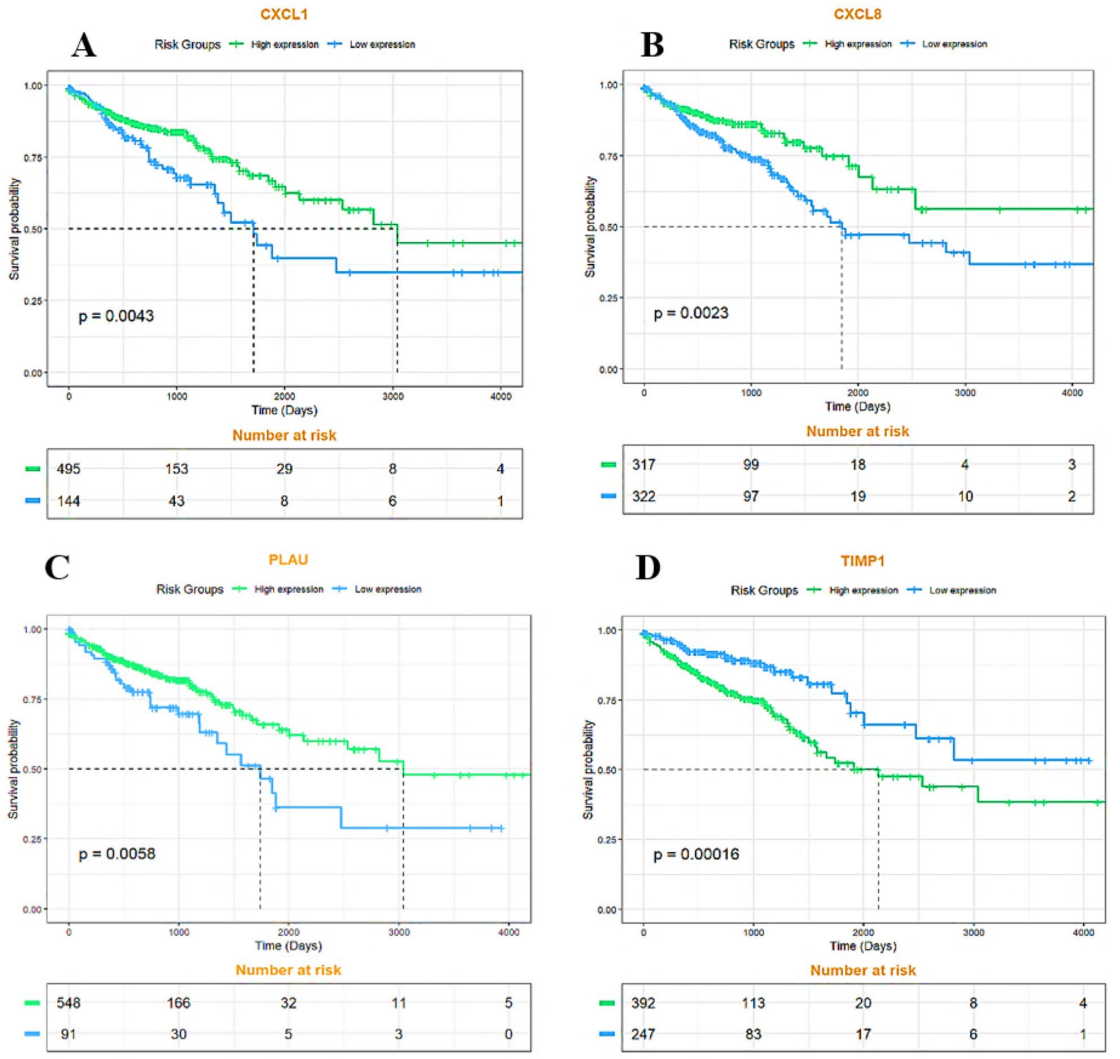
Kaplan–Meier plot of survival analysis of central genes. Survival analysis for the four mentioned hub genes was significant *p* value < 0.05. (**A**) CXCL1 gene (**B**) CXCL8 gene (**C**) PLAU gene (**D**) TIMP gene.

**Figure 13:**
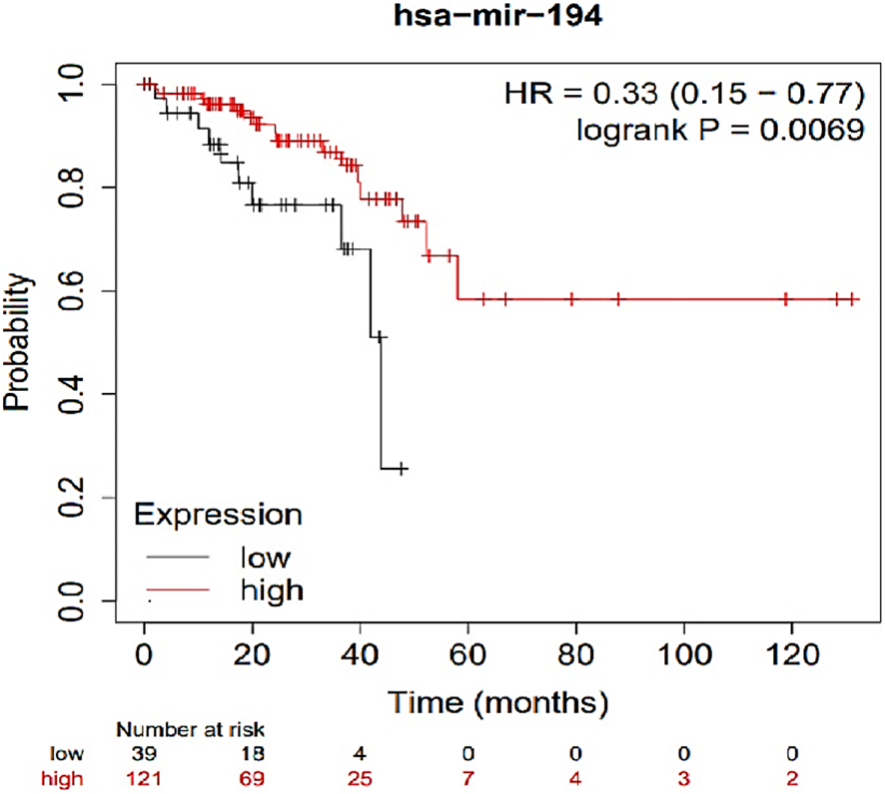
Kaplan–Meier graph of survival analysis has-mir-194. The decrease in the expression of this mir, in proportion to the increase in its expression, decreases the probability of survival more strongly. and (*p* value < 0.05).

The Kaplan–Meier stratified survival analysis with optimal cut-off value revealed four miRNA and has-miR-194-5p that were significantly associated with poor OS of CRC patients (Figs. 12 and 13).

### CXCL8 and SLC16A9 are significantly upregulated in CRC and UC tissues

**Figure14:**
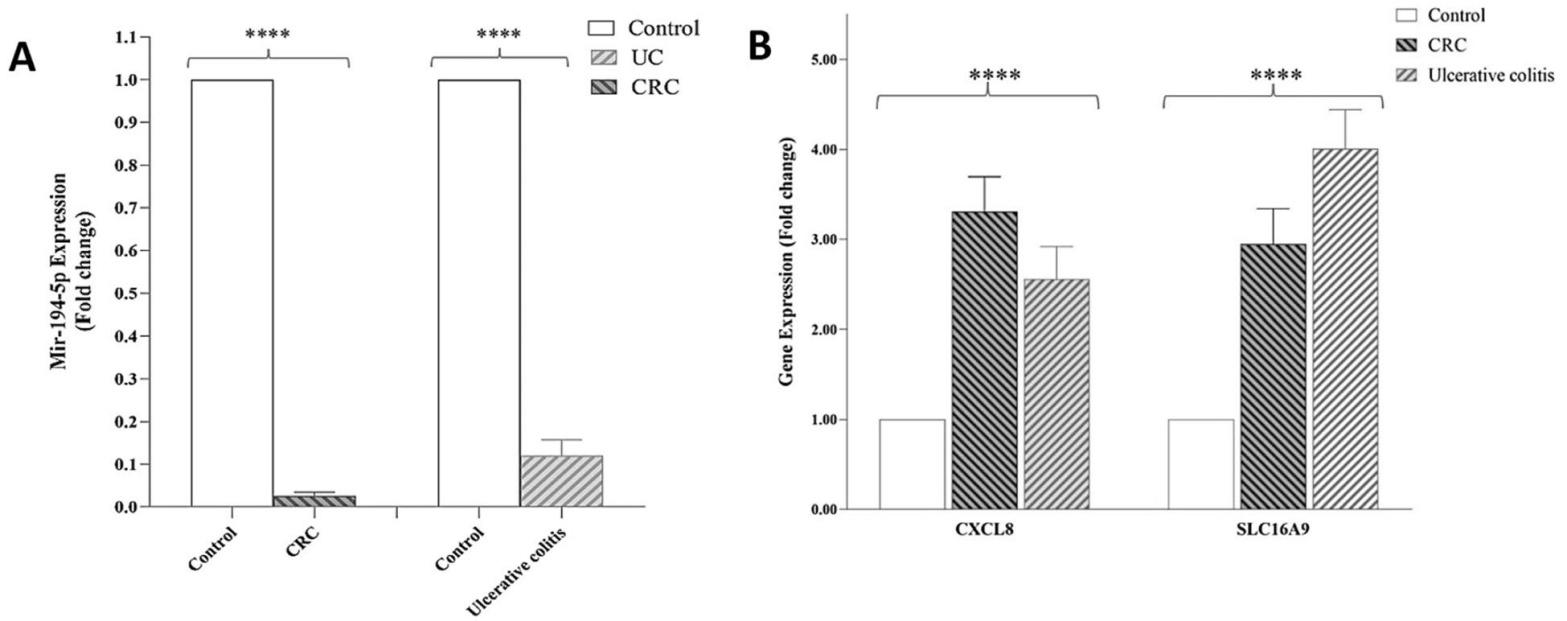
Comparison of biomarker expression based on fold change in CRC, UC, and normal samples. (**A**) miR-194-5p expression, (**B**) CXCL8 and SLCA16A9 gene expression. *P < 0/05, **P < 0/01, ***P < 0/001, ****P < 0/0001, CRC: Colorectal Cancer Cell, Control: Normal tissue, UC: Ulcerative colitis.

Since CXCL8 and SLC16A9 have been shown as a potential and novel therapeutic target with high diagnostic power common to UC and CRC, their expression level in CRC (n = 10), UC (n = 10) and adjacent normal tissues (n = 10) was checked by using RT-qPCR. The level of CXCL8 and SLC16A9 gene expression in cancerous, inflammatory, and normal (control) samples had a significant difference, both genes were significantly higher in CRC and UC tissues than in adjacent normal tissues (Fig. 14). Therefore, in the analysis of TCGA expression, CXCL8 and SLC16A9 were also decreased in CRC compared to normal tissues.

### has-miR-194-5p is significantly downregulated in CRC and UC tissues

The result of comparing the expression of miRNA-194-5p among the three types of intestinal tissue also had a significant difference. Our results show that the expression of miRNA-194-5p in the CRC tissue sample had a significant decrease compared to the control sample. Also, the expression of this miRNA in the tissue sample of IBD has decreased compared to the healthy sample. The comparison of gene and miRNA expression between 3 tissue samples is shown in Fig. 14.

## Discussion

In recent years, finding molecular markers involved in the progression of cancer in humans has created a fascinating yet challenging field of research^31,32^. However, there are problems, such as the lack of more in-depth studies comparing precancerous stages to different stages of cancer with healthy subjects^33^. Therefore, finding biomarkers based on cancer risk-promoting diseases, such as inflammatory diseases, can be very effective in cancer prognosis and treatment in the early stages. In the present study, to discover very important miRNA and mRNA markers that are involved in the process of progression from UC to different stages of CRC. The raw data of microarray-type expression profiles associated with the differential expression of miRNA and mRNA for different stages in CRC and UC disease were used. Our study includes five microarray data sets related to the differential expression of miRNA and mRNA, including tumor tissue samples with different stages of CRC disease, healthy tissue, as well as healthy inflammatory tissue of UC patients in the form of a dataset from the GEO database with the mentioned accession numbers. Finally, these different analyses were merged with the most important miRNAs and mRNAs that are common in all stages of progression from inflammation toward CRC to identify downstream analyses. The analysis identified 98 common DEGs. Then, different target prediction databases were used to find the crucial targets of eight miRNAs that were common in all stages of progression from inflammation to CRC. Among the selected targets (98 targets), the criteria mentioned in the process of inflammation progression toward CRC were introduced as central miRNA-mRNA interactions. Eventually, six important key, and common miRNAs were introduced in the carcinogenesis process. They are including hsa-miR-194-5p, hsa-miR-150-5p, hsa-miR-375-3p, hsa-miR-378a-5p, hsa-miR-342-3p and hsa-miR-141-3p.

GSEA showed that the common DEGs are mainly related to growth signaling pathways, cell proliferation and cycle regulation, migration and metastasis, cell survival, and angiogenesis in CRC, which are the pathways emphasized by many studies in the last few years. For example, one of these pathways suggests the association of DEGs in CRC as targets for EF2 (Eukaryotic elongation factor 2). There are several studies that show the EF2 signaling pathway in the tumorigenesis and progression of CRC^34–38^. As, a systematic research in 2021 has shown the potential role of E2F family members in colon cancer^34^. Also, in the enrichment analysis for UC, common DEGs are mainly associated with inflammatory pathways, epithelial-mesenchymal transition, response to interferon gamma (IFN-γ), and angiogenesis in UC. Studies related to inflammatory bowel diseases have also confirmed this issue.

 For example of these studies, a review on IBD found that in IBD-related intestinal fibrosis, epithelial-mesenchymal transition pathways may serve as a source of new fibroblasts, resulting in the production of too much extracellular matrix^39^. In another study by Victoria Langer in 2019, it was shown that IFN-γ increases the permeability of intestinal vessels by disrupting VE-cadherin connections, which is associated with increased inflammation and the progression of IBD^40^.

In 2019, a study on this inflammatory factor showed that IFN-γ causes pathogenicity and inflammation by breaking the vascular barrier by disrupting the adhesion-binding protein VE-cadherin^40^. Considering the altered expression of miRNAs in different stages of CRC and UC, identifying miRNAs with important interactions with genes involved in the progression of the disease can be very effective in the early diagnosis. In this regard, to identify common miRNAs between CRC and UC, a bipartite miRNA-mRNA interactive network was constructed with genes related to disease progression, in addition to identifying important, key, and central DEGs in CRC and UC, to identify miRNAs that regulate more genes. Especially central hub genes are involved and play key roles in biological processes. Different algorithms were used to find the most important genes based on several network parameters. These parameters include MCODE and Cytohubba with specific parameters such as Degree, Maximal Clique Centrality (MCC), maximum neighborhood component (MNC), Betweenness and Closeness. Also, using the MCODE algorithm, which finds the nodes with the highest interconnectivity in clusters, the central genes and miRNAs in the networks were identified. In this case, the top six nodes shared between the three network parameters of centrality, degree, MCC, MNC, betweenness, interconnectivity, and closeness were chosen as the final goals. These six hub genes include CXCL8, CXCL1, SLC16A9, PLAU, TIMP1 and MMP7. According to the network analysis, CXCL8 has a high degree among hub genes, so it was selected for molecular tests on clinical samples. In the present study, we also analyzed the miRNA-mRNA co-expression network for miRNAs involved in the progression from the initial stages of intestinal inflammation to CRC. We found that among the six selected miRNAs, hsa-miR-150-5p, hsa-miR-375- 3p, in the network, have the most interaction with peripheral DEGs in the interaction network of CRC and UC. Also, miRNAs hsa-miR-378a-5p and hsa-miR-194-5p interact with the centrality of the interactive network, respectively, and regulate high-order nodes and modules, or in other words, they have a hub gene (SLC16A9) relationship. Considering the interaction of hsa-miR-194-5p with the central gene and its importance in CRC according to previous studies, it was selected for investigation on clinical samples. Also, the critical role of this miRNA in growth regulating and proliferation of CRC has been investigated in recent studies. For example, in a study in 2020, it was identified that microRNA-194 could predict colon cancer proliferation through Phospho S6 modulation. Evaluation of the microRNA-194 expression level in clinical samples through RT-qPCR, showed decrease levels in patients with advanced colon cancer^40^. Also, in a comprehensive study on HMGA2 and regulating microRNAs in CRC determined miR-194 is as important as HMGA2 and both coordinately regulate CRC. It was also concluded that VAPA (Vesicle-Associated Membrane Protein-Associated Protein A) is a direct target for miR-194. In this regard, in vitro experiments on several cell lines revealed that miR-194 ultimately inhibits CRC cell proliferation. Also, overexpression of the tumor suppressor miR-194 sensitizes CRC cells to chemotherapy^41^. In a 2019 study by Sun B et al., the effect of miR-194 on the proliferation of colon cancer stem cells was determined. Specifically, miR-194 inhibits CRC stem cell proliferation and promotes CRC stem cell apoptosis. It is involved by directly targeting SSH2. In other words, they showed for the first time that the expression of miR-194 is decreased in CRC stem cells. In addition, overexpression of miR-194 led to the blocking of G1/S transition, induction of cell apoptosis process, and thus suppression of malignant behavior of CRC stem cells^42^. Also, in a study on the biological effects, mechanisms, and clinical significance of miR-194 in mucosal, tissue samples, and in vivo and in vitro clinical conditions. Functional assays showed that overexpression of miR-194 inhibited the survival and invasion of CRC cells in vitro and induced tumor suppression in vivo. Overall, their data showed that miR-194 acts as a tumor suppressor in colorectal carcinogenesis by targeting the PDK1/AKT2/XIAP pathway and could be an important diagnostic and prognostic biomarker for CRC^43^. Zhan-long Shen et al. showed that the downregulation of miR-194 expression was associated with tumor size, and tumor differentiation as well as TNM stage^44^. The Kaplan–Meier and multivariate survival analysis showed that miR-194 was downregulated and correlated with overall survival. Furthermore, functional assays showed that overexpression of miR-194 in CRC cell lines inhibited cell proliferation both in vitro and in vivo. Their results showed that miR-194, a regulator of the MAP4K4/c-Jun/MDM2 signaling pathway, can act as a tumor suppressor and introduced it as a new target for the prevention and treatment of CRC^44^. Also, in a longitudinal study on different models of UC in mice, a serum miRNA signature was identified, which indicated the development of colitis. The validated colitis signature consists of nine miRNAs: miR-29b-3p, -122-5p, -mir-192-5p, mir-194-5p, -375-3p, mir-150-5p, and -146a-3p, miR-148a-3p and -199a-3p. For confirmation, qPCR was used to analyze serum expression levels^45^. Also, during a genome-wide study using next-generation sequencing, Jingmei Lin and colleagues identified new microRNA biomarkers for the differential diagnosis of inflammatory bowel diseases (UC from CD). Illumina; next-generation sequencing on freshly frozen non-dysplastic colonic mucosa of the most distal colectomy and control group and RT-qPCR validity assay performed on frozen tissue from UC (n = 20) and CD (n = 10) samples, specific expression of miR- 147b confirmed miR-194, miR-383, miR-615, and miR^45^. In this regard, our research also confirms the reduction of miR-194 expression and its role in improving the intestinal inflammatory process. Regarding the role of miR-378a-5p in IBDs, a study conducted by Quaglio et al. in 2021 on 20 clonal tissues obtained from Crohn’s and UC patients showed increased expression of miR-378a-3p/5p compared to the colonic tissue of UC, and this increased expression could be a mechanism leading to the increased possibility of CRC development^27^. GO, and KEGG functional analyzes were considered to investigate, and analyze the biological processes and pathways in which hub genes shared between CRC and UC disease are involved. This analysis showed that hub genes were significantly enriched in specific cellular components. The results of GO analysis showed that these hub genes are related to cancer purity and immune penetration of different cells in CRC. They have a strong diagnostic value for UC and CRC. Also, the analysis of KEGG biological pathways shows that the genes involved in the stages of inflammation and cancer are involved in collagen synthesis, intercellular connections, cell growth and proliferation, and the regulation of miRNAs, which are potentially involved in cancer progression. In this regard, the genes CXCl8, CXCL1, and CXCL2 are members of the CXCL family (C-X-C chemokine motif ligand), MMP7, MMP3 and MMP1 are members of the matrix metalloproteinases (MMPs) and TIMP1, which were mainly shared in these pathways. In several studies, TIMP1 expression was increased in cancer patients^46–48^. This phenomenon is also true in our study. In-depth studies have shown that TIMP1 accelerates cell proliferation by activating YAP/TAZ in cancer, suggesting that the TIMP1-YAP/TAZ axis may be a potential new drug target for treating cancer patients. In addition, studies have also shown that TIMP1 is an important marker of UC^49^ and CRA^50^. Regarding the role of these markers in CRC, they were also analyzed in recent studies as therapeutic targets and important biomarkers. CXCL1 (C-X-C motif chemokine ligand 3) and CXCL8 (C-X-C motif chemokine ligand 8) were upregulated in CRC patients, which is consistent with previous studies^49–51^. Similar; findings were presented by Baier et al., who reported significantly higher levels of CXCL1 in CRC tissue compared to normal mucosa. It was concluded that this chemokine contributes to tumor growth^52^ which was confirmed by Bandpalli et al.^53^. In addition, CXCL1-CXCR2 receptor expression was also increased in human CRC^54^. A study by Zhuo et al. also confirmed that CXCL1 expression was higher in CRC tissue than controls and was significantly correlated with tumor diameter, T stage, N stage, M stage, lymphatic vessel invasion, and CEA levels^55^. About CXCL8, our bioinformatics results, and molecular tests on tissue samples indicate an increase in the expression of this gene in tumor and inflammation samples. Some studies validate our result. They show that CXCL8 and their cognate receptors, can mediate tumor growth, proliferation, survival, neo angiogenesis, and metastasis of malignant cells, including CRC. For example, in some study CXCL8 expression was assessed by immunohistochemistry. They showed that CXCL8 was significantly higher in all CRC tissue samples compared to non-malignant samples^56,57^, It was also confirmed by a study in 2020 by Jie Li et al.^51^. In addition, lack of CXCL8 expression in epithelial cells may be a factor for good prognosis^58–61^. In addition, our results show that the increased expression of this gene is higher in cancer samples than in UC. So, it shows its importance as a marker in the CRC progression due to its higher expression in cancer samples. It should also be mentioned that in UC disease, it has been determined only during a limited study that CXCL8 participates in the pathogenesis of this disease^61^.

During the GO-KEGG analysis in UC disease stages, it was found that genes were generally highly related in pathways related to the immune system, inflammatory responses, proteolysis, and cell migration. Among these, the genes of matrix metalloproteinases (MMPs), and the genes of the family of solute carrier proteins (SLCs) are of great importance. The SLC gene family is generally enriched in the plasma membrane and plays a role in epithelial permeability and barrier function in the intestine^62^. In this regard, an article was published by Amir Shaghaghi et al. They showed that the SLC family gene is a new glucose/ dehydroascorbic acid (DHA) transporter GLUT14 encodes, is associated with inflammatory bowel disease^63^. In a study by Bayan et al., SLC4A4 (solute carrier family 4 member 4) and CEACAM7 (carcinoembryonic antigen-associated cell adhesion molecule 7) were found to be associated with poor prognosis in CRC^64^. Currently, there is not any research on the increased expression of SLC16A9 associated with intestinal diseases. Studies related to SLC16A9 have usually included investigations of hematological and metabolic disorders such as gout^60^. Few studies have been conducted investigating the relationship between the expression of this gene in another cancers. Based on our results, SLC16A9 is shared gene in CRC and UC disease, and affected by two important DEmiRNAs, mir-194-5p and miR-378a-5. It was expected that this gene would be upregulated as a DEG in molecular evaluation. In this regard, RT-qPCR analysis on tumor and inflammation samples compared to the control confirmed this issue. The increased expression of this marker in UC samples compared to CRC can be explained because of its critical role in defense and inflammatory mechanisms. As mentioned earlier, this gene family plays a role in epithelial permeability and barrier function in the intestine^62^. where the natural microenvironment of the intestine is still trying to maintain homeostasis and optimize conditions. But its increased expression in tumor conditions may sound the alarm in the stability of activity and upregulation of this gene. In other words, upregulated regulation of this gene in both inflammatory conditions and CRC may be a factor involved in the development and progression of CACC.

The above studies and our research show that the six identified hub genes are likely to be critical factors in the development and progression of cancer and CRC. At the same time, our research shows that CXCL1 and CXCL8 as hub gene with high degree and effect; Also, mir-194-5p and miR-378a-5p, which target and regulate the central hub gene SLC16A9, have continuous changes in the progression of UC towards CRC, and all of them have diagnostic value. Analysis by RT-qPCR method has confirmed this issue approved. Also, we speculate that SLC16A9, which was identified as a target of mir-194-5p and miR-378a-5p, is an important factor in the development of intestinal inflammation towards CRC.As we introduce the hsa-mir-194-5p and hsa-mir-378a-5p as key regulators of this pathway.

## Conclusion

According to this research, we identified six central genes including, CXCL1, CXCL8, MMP 7, SLC16A9, PLAY, and TIMP 1, which are upregulated and have clinical diagnostic value for UC and CRC. Also, the SLC16A9 gene is negatively regulated by mir-194-5p and miR-378a-5p. Among these, central genes CXCL1, CXCL8, MMP7, PLAU, and TIMP1 are related to CRC. Importantly, by examining the expression of the CXCL8 gene, miRNA-194-5p, and its target gene (SLC16A9) on intestinal cancerous and inflammatory tissue samples by RT-qPCR method, we found that two new genes, CXCL8, SLC16A9 with increased expression in cancerous and inflammatory samples, they have a potential diagnostic value to indicate the occurrence of CRC and are related to the prognosis of CRC, and they are critical and valuable for the progression of UC to colorectal adenoma and colorectal cancer and should be confirmed in future studies. According to the bioinformatics and RT-qPCR findings, mir-194-5p also has a tumor suppressive regulatory role by reducing its expression more in CRC than in UC. Therefore, it can be concluded that miR-378a/SLCA16A9 or miR-194/SLC16A9 can be potential biomarkers for predicting treatment response. They be considered as therapeutic targets. Although future studies are needed for the final confirmation of these factors.

## Data Availability

The datasets generated and/or analyzed during the current study are available in the [https://www.ncbi.nlm.nih.gov/geo/database] and https://portal.gdc.cancer.gov/]. The original contributions presented in the study are included in the article/Supplementary Material, further inquiries are available from the corresponding author on reasonable request.
